# Supplementary material for: Identification of photoperiod-induced specific miRNAs in the adrenal glands of Sunite sheep (Ovis aries)
Source: Front Vet Sci. 2022 Jul 22;9:888207. doi: 10.3389/fvets.2022.888207 (PMC9354845; doi:10.3389/fvets.2022.888207)
Supplement: Supplementary file 1 [file Data_Sheet_1.ZIP › Supplementary file/miRDeep2 file.zip!/miRDeep2 file/Novel_Structure/LP42A3/Novel_230.pdf]

Provisional ID	:	NC_019474.2_18029
Score total	:	5.4
Score for star read(s)	:	3.9
Score for read counts	:	-1.8
Score for mfe	:	1.8
Score for randfold	:	1.6
Score for cons. seed	:	
Total read count	:	8
Mature read count	:	7
Loop read count	:	0
Star read count	:	1

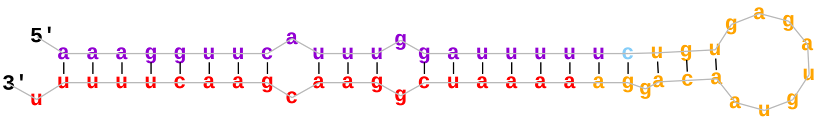

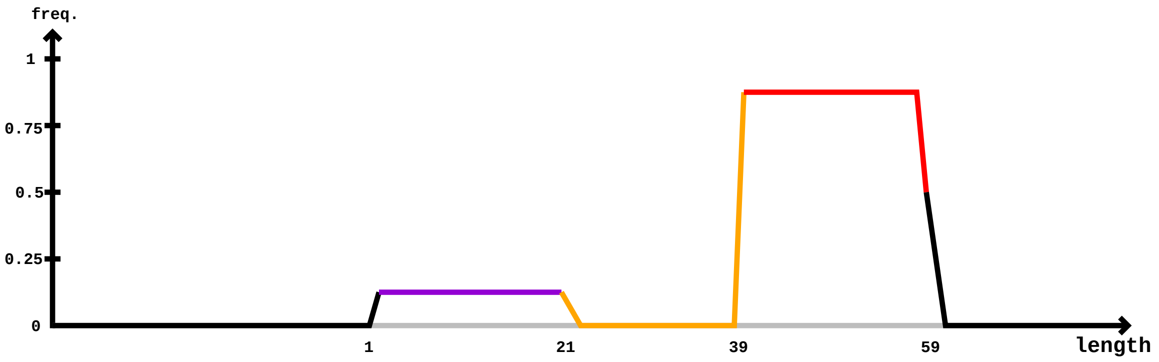

Star **Mature**

```
5'- cagcuguauggauuuucguauugguuuggccuaaagguucauuuggauuuuucugugagauguaacaggaaaaaucggaacgaacuuuuuagccaacccaaugcaaacag   -3'   obs
    cagcuguauggauuuucguauugguuuggccuaaagguucauuuggauuuuucugugagauguaacaggaaaaaucggaacgaacuuuuuagccaacccaaugcaaacag         exp
    .................(((((((.(((((..((((((((.(((.(((((((((((........))).)))))))).))).))))))))..))))).)))))))......    reads   mm   sample
    ................................aaagguucauuuggGuuuuu..........................................................    1       1    seq
    ......................................................................aaaaucUgaacgaacuuuu.....................    3       1    seq
    ......................................................................aaaaucggaacgaacuuuuu....................    4       0    seq
```
